# Supplementary material for: Enhancing effect of 5-azacytidine on saline–alkaline resistance of Akebia trifoliata and underlying physiological and transcriptomic mechanisms
Source: PeerJ. 2025 May 14;13:e19285. doi: 10.7717/peerj.19285 (PMC12085116; doi:10.7717/peerj.19285)
Supplement: Supplemental Information 1 — Differential expression results of Plant hormone signal transduction. [file peerj-13-19285-s001.doc]

**Table S1** **Differential expression results of** Plant hormone signal transduction

| gene name | Gene ID | KO name | Salt vs Con | | Salt + 5-AzaC vs Con | | Salt + 5-AzaC vs Salt | |
| --- | --- | --- | --- | --- | --- | --- | --- | --- |
|  |  |  | Log_2_FC | FDR | Log_2_FC | FDR | Log_2_FC | FDR |
| auxin transporter-like protein 3 | TRINITY_DN2890_c0_g1 | AUX1, LAX | -1.9632297097 | 1.18330359345E-14 | -1.91045576859 | 3.42568218254E-9 | 0.0309779864897 | 0.964239502676 |
| auxin influx carrier component | TRINITY_DN34522_c0_g2 | AUX1, LAX | -3.964038186 | 0.00530733840612 | -3.097519218 | 0.681958707609 | 0.824791726 | 0.0903526538073 |
| Amino acid transporter | TRINITY_DN947_c0_g1 | AUX1, LAX | -1.586635155 | 5.90448893159E-12 | -1.442404178 | 2.73078641686E-7 | 0.122153435 | 0.79961305436 |
| GH3 auxin-responsive promoter | TRINITY_DN16410_c0_g1 | GH3 | 5.975768278 | 0.316386182472 | 6.762785351 | 0.008237922 | 0.847967805 | 1 |
| GH3 auxin-responsive promoter | TRINITY_DN5101_c0_g1 | GH3 | 1.94525491 | 7.60409586545E-23 | 1.006319824 | 0.00530406544815 | -0.956492798 | 0.000430437520194 |
| hypothetical protein | TRINITY_DN1146_c0_g3 | IAA | -1.929085472 | 2.06389170553E-20 | -2.050184931 | 2.66736926854E-8 | -0.141876849 | 0.810033468376 |
| hypothetical protein IFM89 | TRINITY_DN1146_c0_g4 | IAA | -2.934266561 | 2.99290387286E-32 | -2.081190837 | 8.3613264249E-10 | 0.828750281 | 0.0279573449688 |
| AUX/IAA protein | TRINITY_DN12620_c1_g1 | IAA | -2.1154635 | 0.00050918144745 | -3.317165943 | 1.10259089537E-5 | -1.223632991 | 0.269005068463 |
| indole-3-acetic acid 7 | TRINITY_DN14490_c0_g2 | IAA | 4.406418049 | 1.21526596219E-25 | 2.333642541 | 0.309870942984 | -2.087610235 | 0.296051090074 |
| hypothetical protein | TRINITY_DN1493_c0_g3 | IAA | -1.053431883 | 7.08643981424E-6 | -0.967113876 | 0.000536782809371 | 0.068196155 | 0.861674285878 |
| hypothetical protein HHK36 | TRINITY_DN27928_c0_g1 | IAA | -3.735889034 | 0.00106106133985 | -2.637665939 | 0.0790842835752 | 1.08101087 | 0.710048861328 |
| auxin-responsive protein IAA1 | TRINITY_DN32289_c0_g2 | IAA | -2.556678411 | 5.17464416099E-7 | -1.236729002 | 0.0344417313167 | 1.294593632 | 0.0611484141072 |
| hypothetical protein | TRINITY_DN49469_c0_g1 | IAA | -6.68651252 | 0.000853449631888 | -2.458865938 | 0.333118403801 | 4.185339699 | 1 |
| Auxin-responsive protein | TRINITY_DN5394_c1_g1 | IAA | -0.013802991 | 0.976539583421 | 1.027764391 | 0.007069989 | 1.027308135 | 0.000740983 |
| auxin-responsive protein IAA4-like isoform X1 | TRINITY_DN54_c0_g1 | IAA | 2.788720125 | 2.58183428222E-7 | 2.766432608 | 8.88857165577E-6 | -0.038763931 | 0.968305743563 |
| auxin-induced | TRINITY_DN5683_c0_g1 | IAA | -2.378786289 | 6.75307004159E-26 | -2.362714156 | 0.0313686191724 | -0.008798186 | 0.993668714982 |
| Hypothetical protein | TRINITY_DN8068_c0_g1 | IAA | -6.121570862 | 1.05735954332E-15 | -3.53812187 | 0.0409954110902 | 2.547266943 | 0.0202371077471 |
| hypothetical protein | TRINITY_DN8068_c0_g3 | IAA | -2.959286733 | 4.95401326019E-5 | -2.318966647 | 0.00269061234833 | 0.594635195 | 0.730832947097 |
| auxin-responsive protein IAA25-like protein | TRINITY_DN8281_c0_g1 | IAA | -1.595163919 | 5.60711757877E-5 | -0.821297837 | 0.105083579001 | 0.757085478 | 0.165732573767 |
| hypothetical protein | TRINITY_DN14422_c0_g1 | K14486, ARF | -1.826382457 | 0.00256962432299 | -0.930993421 | 0.185125581911 | 0.877614629 | 0.288390713064 |
| Auxin response factor 5 | TRINITY_DN38304_c1_g1 | K14486, ARF | -5.854604733 | 0.00152216838025 | -2.416099452 | 0.0990754977732 | 3.440752442 | 1 |
| hypothetical protein | TRINITY_DN5433_c0_g1 | K14486, ARF | -1.061384344 | 0.0103374296422 | -1.228883561 | 0.0481462307282 | -0.186969153 | 0.826228932296 |
| hypothetical protein | TRINITY_DN10566_c0_g1 | SAUR | -6.20065722 | 0.00359389269725 | -2.170800883 | 0.274031426453 | 3.990710235 | 1 |
| hypothetical protein | TRINITY_DN10828_c0_g1 | SAUR | 1.590796705 | 1.28922014201E-8 | 1.133800562 | 0.00161192584797 | -0.467259545 | 0.148800202313 |
| Auxin-responsive protein | TRINITY_DN11786_c0_g1 | SAUR | -1.426494778 | 0.197100203453 | 0.423576551 | 0.699281835 | 1.822004939 | 0.031883437 |
| auxin-responsive protein | TRINITY_DN12560_c0_g2 | SAUR | -4.194447741 | 0.000204047886116 | -2.578596272 | 0.0714277566785 | 1.583302677 | 0.529221110247 |
| auxin-responsive protein | TRINITY_DN12560_c0_g3 | SAUR | -6.469197795 | 0.00011280299543 | -4.789941581 | 0.00189272921443 | 1.621169748 | 1 |
| hypothetical protein | TRINITY_DN12560_c0_g4 | SAUR | -5.344386391 | 0.000703042822023 | -4.172599967 | 0.00654170828253 | 1.083970506 | 1 |
| hypothetical protein | TRINITY_DN13178_c1_g1 | SAUR | -4.358339944 | 0.0340205838701 | -4.155290553 | 0.0752874316152 | 0.158517682 | 1 |
| hypothetical protein | TRINITY_DN14549_c0_g1 | SAUR | -3.344433869 | 0.0117091065547 | -2.56476451 | 0.126799212539 | 0.747640129 | 0.840649144473 |
| auxin-induced protein 15A | TRINITY_DN15510_c0_g1 | SAUR | 3.017481295 | 0.0230986542288 | 2.126754955 | 0.247309434512 | -0.903435163 | 0.425376296247 |
| auxin-responsive protein | TRINITY_DN15549_c0_g1 | SAUR | -5.784703611 | 9.23006670049E-5 | -4.608578794 | 0.000604277209175 | 1.063734774 | 1 |
| auxin-induced protein X10A | TRINITY_DN15549_c0_g2 | SAUR | -7.005827749 | 7.08290113566E-6 | -3.752738803 | 0.00220671478062 | 3.203274862 | 1 |
| hypothetical protein | TRINITY_DN17666_c1_g1 | SAUR | -3.651102642 | 0.00380745896802 | -4.061704428 | 0.0078496210807 | -0.469244246 | 1 |
| hypothetical protein | TRINITY_DN17666_c1_g2 | SAUR | -6.139153949 | 0.00457052590658 | -4.350881028 | 3.45781236177E-6 | 1.74603219 | 1 |
| hypothetical protein | TRINITY_DN21872_c0_g2 | SAUR | -5.665467098 | 0.0236377969232 | -0.743020137 | 0.786591377426 | 4.916759344 | 1 |
| hypothetical protein | TRINITY_DN21872_c1_g1 | SAUR | -1.194770944 | 4.1319555438E-5 | -0.364304182 | 0.508392160661 | 0.813114098 | 0.0464339870789 |
| hypothetical protein | TRINITY_DN24189_c0_g1 | SAUR | -6.932850416 | 8.16524522241E-6 | -4.525317614 | 0.000203983430207 | 2.352251521 | 1 |
| hypothetical protein | TRINITY_DN302_c0_g2 | SAUR | -4.731167012 | 6.06117192958E-48 | -2.126103843 | 0.354037335353 | 2.576555265 | 0.150879907306 |
| Auxin-induced protein | TRINITY_DN302_c0_g3 | SAUR | -5.250632031 | 1.7391901259E-18 | -2.899546884 | 3.68478999641E-6 | 2.308001013 | 0.0186844533075 |
| hypothetical protein | TRINITY_DN302_c0_g4 | SAUR | -4.526508325 | 3.74438036955E-20 | -1.94555986 | 0.00137119330251 | 2.548448411 | 0.0001094419381 |
| hypothetical protein | TRINITY_DN33812_c0_g1 | SAUR | -4.604002735 | 4.73028817305E-10 | -3.371035365 | 0.000221363077863 | 1.181984466 | 0.541071710024 |
| Indole-3-acetic acid-induced protein | TRINITY_DN340_c0_g1 | SAUR | 0.718856806 | 0.32236319012 | -0.939458539 | 0.429910237 | -1.670735594 | 0.017593043 |
| hypothetical protein | TRINITY_DN36765_c0_g1 | SAUR | -4.328424338 | 0.0349886770167 | -0.969936131 | 0.705841614475 | 3.290673801 | 0.307168564404 |
| Auxin-induced protein | TRINITY_DN36765_c0_g2 | SAUR | -3.418551566 | 5.56812832935E-8 | -1.956841811 | 0.103393055854 | 1.442253905 | 0.349308973789 |
| hypothetical protein | TRINITY_DN4129_c0_g5 | SAUR | -5.631287242 | 0.000274010823684 | -3.420118074 | 0.0282425689559 | 2.17943828 | 1 |
| hypothetical protein | TRINITY_DN4129_c1_g1 | SAUR | -5.549201837 | 1.1614160299E-8 | -2.305854692 | 0.412007994203 | 3.210461019 | 0.0903760286675 |
| hypothetical protein | TRINITY_DN4210_c0_g2 | SAUR | -6.758931623 | 3.4393935785E-5 | -2.593902652 | 0.0824707712772 | 4.122105206 | 0.177237507261 |
| hypothetical protein | TRINITY_DN4210_c0_g3 | SAUR | -4.12067531 | 1.15676902618E-17 | -3.036161388 | 6.99003289115E-6 | 1.060098217 | 0.371992368262 |
| hypothetical protein | TRINITY_DN4210_c0_g4 | SAUR | -5.015909678 | 5.0759491915E-9 | -3.071948922 | 0.237029538176 | 1.939002171 | 0.467465876882 |
| hypothetical protein | TRINITY_DN4210_c2_g1 | SAUR | -5.596473624 | 6.00670077278E-23 | -3.374125656 | 0.0289634256204 | 2.198400423 | 0.107910959181 |
| hypothetical protein | TRINITY_DN4210_c2_g2 | SAUR | -5.67931808 | 0.0253122708725 | -2.620680916 | 0.373739046009 | 3.016129591 | 1 |
| hypothetical protein | TRINITY_DN45537_c0_g1 | SAUR | -7.267607783 | 2.84667329816E-6 | -3.862445471 | 0.00179751625956 | 3.35054746 | 1 |
| hypothetical protein | TRINITY_DN46347_c0_g1 | SAUR | -5.455438605 | 4.75149291806E-7 | -1.876163908 | 0.145493189803 | 3.546818593 | 0.0374469440735 |
| hypothetical protein | TRINITY_DN46347_c0_g2 | SAUR | -4.875972726 | 0.036190441379 | -2.802654497 | 0.204931593933 | 2.024404829 | 1 |
| hypothetical protein | TRINITY_DN4666_c0_g1 | SAUR | -4.291197711 | 1.71959296485E-49 | -2.433806511 | 0.125551325337 | 1.827162046 | 0.188750246913 |
| auxin-induced protein 15A | TRINITY_DN4666_c2_g1 | SAUR | -6.307385438 | 2.23228873502E-15 | -3.798026014 | 4.083579835E-5 | 2.472462621 | 0.210862394233 |
| auxin-responsive protein | TRINITY_DN5283_c0_g1 | SAUR | -4.804213351 |  | -2.86673651 |  | 1.906098382 |  |
| hypothetical protein | TRINITY_DN5283_c1_g1 | SAUR | -5.651594209 | 0.0235687643333 | -3.025712838 | 0.206706162531 | 2.579401288 | 1 |
| auxin-responsive protein | TRINITY_DN5928_c2_g1 | SAUR | -1.311835379 | 9.87131291942E-8 | -0.554313449 | 0.126614193414 | 0.741039826 | 0.0180651286436 |
| auxin-responsive protein | TRINITY_DN5928_c2_g2 | SAUR | -1.243309392 | 0.000357829148684 | -0.877873682 | 0.0364633627244 | 0.354166616 | 0.46723315642 |
| hypothetical protein | TRINITY_DN7510_c0_g1 | SAUR | -5.939899952 | 3.2243693269E-48 | -2.83184005 | 0.140665520434 | 3.084865898 | 0.0022118687626 |
| hypothetical protein | TRINITY_DN7510_c0_g3 | SAUR | -5.531018658 | 1.07795270472E-43 | -2.99928331 | 0.104508559844 | 2.501625249 | 0.0207881074629 |
| auxin-responsive protein | TRINITY_DN8225_c0_g1 | SAUR | -8.38718972 | 1.04113509919E-8 | -3.260676063 | 0.461769885002 | 5.102445799 | 1 |
| hypothetical protein | TRINITY_DN8828_c0_g1 | SAUR | -6.008740058 | 3.42406143807E-22 | -3.250935341 | 0.000126395936344 | 2.706195886 | 0.0514451362043 |
| hypothetical protein | TRINITY_DN8828_c0_g2 | SAUR | -4.784271893 | 0.00141075359896 | -3.033750995 | 0.0747189643261 | 1.669997221 | 1 |
| auxin-responsive protein | TRINITY_DN9402_c0_g1 | SAUR | -1.760504189 | 5.94435460617E-12 | -1.002501181 | 0.000482409952761 | 0.738288057 | 0.004601047468 |
| hypothetical protein | TRINITY_DN9529_c0_g1 | SAUR | -7.057497171 | 0.000108383115691 | -3.655669175 | 0.0373054829217 | 3.37520097 | 1 |
| Transport inhibitor response 1 protein | TRINITY_DN162_c0_g1 | TIR1 | -1.238204201 | 0.0000392742195684 | -1.622617125 | 0.000662444 | -0.40724781 | 0.427548888 |

Con is control, Salt (150 mmol/L Na+), and Salt+5-AzaC (200 μmol/L 5-AzaC + 150 mmol/L Na+), FDR<0.05000 indicates significant.
