# Supplementary material for: Enhancing effect of 5-azacytidine on saline–alkaline resistance of Akebia trifoliata and underlying physiological and transcriptomic mechanisms
Source: PeerJ. 2025 May 14;13:e19285. doi: 10.7717/peerj.19285 (PMC12085116; doi:10.7717/peerj.19285)
Supplement: Supplemental Information 2 [file peerj-13-19285-s002.doc]

**Table S2 Differential expression results of Phenylpropanoid biosynthesis**

| gene name | Gene ID | KO name | Salt vs Con | | Salt + 5-AzaC vs Con | | Salt + 5-AzaC vs Salt | |
| --- | --- | --- | --- | --- | --- | --- | --- | --- |
|  |  |  | Log_2_FC | FDR | Log_2_FC | FDR | Log_2_FC | FDR |
| AMP-dependent synthetase/ligase | TRINITY_DN3010_c0_g1 | 4CL | -1.286765283 | 1.13854786265E-5 | -1.349189619 | 0.000126395936344 | -0.078749756 | 0.880250227889 |
| AMP-dependent synthetase/ligase | TRINITY_DN6781_c0_g1 | 4CL | 1.154484807 | 3.39341292892E-5 | 1.030940519 | 0.0483122428929 | -0.147252367 | 0.825744292376 |
| 4-coumarate-CoA ligase | TRINITY_DN935_c1_g1 | 4CL | -1.377216518 | 3.09233981634E-12 | -0.539322441 | 0.0238185833627 | 0.822933783 | 2.69265408911E-5 |
| hypothetical protein | TRINITY_DN11725_c0_g1 | bglB | 1.100219133 | 0.0194522189438 | 1.343570421 | 0.0101641700044 | 0.234314369 | 0.603804292534 |
| hypothetical protein | TRINITY_DN19253_c1_g1 | bglB | -1.961485779 | 1.29307835038E-10 | 0.386245369 | 0.498186965445 | 2.33069932 | 7.30976329749E-10 |
| hypothetical protein | TRINITY_DN28204_c0_g2 | bglB | -3.187870808 | 3.79760274375E-15 | -1.286525645 | 0.0220595617266 | 1.867510217 | 0.000165175109413 |
| hypothetical protein | TRINITY_DN349_c0_g1 | bglB | 1.074022092 | 1.70481917913E-7 | 1.696491547 | 6.9956466812E-16 | 0.609415352 | 0.0020415246217 |
| Glycosyl hydrolase family 1 | TRINITY_DN6044_c2_g1 | bglB | 0.841976746 | 0.032821046 | 1.41554821 | 3.54E-05 | 0.558722635 | 0.084347384 |
| hypothetical protein | TRINITY_DN21688_c0_g1 | bglX | -1.014617332 | 0.00124744755932 | -0.930168966 | 0.0117174101263 | 0.065145238 | 0.907739160356 |
| hypothetical protein | TRINITY_DN6020_c0_g1 | bglX | -3.189003153 | 6.94713183378E-8 | -0.477674698 | 0.392612359218 | 2.680466142 | 3.55321936607E-5 |
| probable mannitol dehydrogenase | TRINITY_DN5158_c0_g2 | CAD | 2.791365398 | 0.0351926418625 | 1.09486209 | 0.654675405535 | -1.70179229 | 0.128459820691 |
| hypothetical protein | TRINITY_DN848_c0_g3 | CAD | -1.249646731 | 1.33575639801E-8 | -0.613188604 | 0.0834143844807 | 0.616443318 | 0.0314370895223 |
| hypothetical protein | TRINITY_DN688_c0_g1 | COMT | -1.620407331 | 5.45237771976E-12 | -0.310077738 | 0.495962093853 | 1.296440883 | 7.60984704067E-5 |
| hypothetical protein | TRINITY_DN2942_c0_g1 | CSE | 2.202038577 | 8.32209807483E-13 | 2.430003683 | 7.96323029665E-8 | 0.228723226 | 0.696677373605 |
| hypothetical protein | TRINITY_DN5257_c0_g2 | CSE;MGLL | -1.47491799 | 0.000355960512688 | -0.804662516 | 0.117198811433 | 0.649634636 | 0.178696386474 |
| hypothetical protein | TRINITY_DN10480_c0_g1 | E1.11.1.7 | -2.372853564 | 0.0174746072749 | -1.776522125 | 3.95698295396E-7 | 0.56648076 | 0.491172091393 |
| Cationic peroxidase 1 | TRINITY_DN11677_c0_g1 | E1.11.1.7 | 1.795420267 | 0.652316973 | 5.846553163 | 0.000171203 | 4.09825275 | 0.174726572 |
| hypothetical protein | TRINITY_DN13780_c0_g1 | E1.11.1.7 | 7.476957671 | 0.0434238595846 | 7.656168952 | 0.00688947176772 | 0.217840699 | 1 |
| Peroxidase | TRINITY_DN14027_c0_g1 | E1.11.1.7 | 3.213977169 | 0.209855931 | 3.791169764 | 0.004955134 | 0.624636817 | 1 |
| hypothetical protein | TRINITY_DN18253_c0_g2 | E1.11.1.7 | -1.258427537 | 5.38860898352E-5 | -1.858632153 | 6.91529877902E-7 | -0.615717774 | 0.192457972007 |
| hypothetical protein | TRINITY_DN18378_c0_g1 | E1.11.1.7 | -6.934318098 | 8.21467160036E-6 | -3.164483203 | 0.00794436691154 | 3.72425535 | 1 |
| peroxidase | TRINITY_DN2067_c0_g1 | E1.11.1.7 | -0.138268446 | 0.814133416 | 1.458165658 | 0.001808937 | 1.578152133 | 0.000240043 |
| Peroxidase | TRINITY_DN21570_c0_g1 | E1.11.1.7 | -2.505491659 | 7.35122411111E-17 | -1.84016127 | 1.87477670267E-6 | 0.63858501 | 0.22363931329 |
| hypothetical protein | TRINITY_DN25115_c0_g1 | E1.11.1.7 | 1.641258591 | 0.00581095531082 | 2.720877291 | 6.60150685453E-6 | 1.076528886 | 0.000720064871782 |
| hypothetical protein | TRINITY_DN2702_c0_g1 | E1.11.1.7 | -4.538465183 | 1.11307251667E-56 | -2.606555371 | 0.0448539644979 | 1.905366135 | 0.00627882505706 |
| hypothetical protein | TRINITY_DN29908_c0_g1 | E1.11.1.7 | 1.50432 | 9.11313500228E-6 | 0.553831182 | 0.376748515908 | -0.962092039 | 0.0112643290312 |
| hypothetical protein | TRINITY_DN3294_c0_g1 | E1.11.1.7 | 2.038606194 | 0.0012188252049 | 1.722465195 | 0.0184545093342 | -0.330574013 | 0.629338325277 |
| peroxidase | TRINITY_DN33946_c0_g1 | E1.11.1.7 | 2.920821244 | 1 | 5.748091586 | 0.015473653 | 2.819954892 | 0.083879324 |
| hypothetical protein | TRINITY_DN3425_c1_g2 | E1.11.1.7 | 2.543972132 | 9.02313342424E-8 | 2.217806873 | 0.000393652008357 | -0.334826567 | 0.569061197606 |
| hypothetical protein | TRINITY_DN34988_c0_g1 | E1.11.1.7 | -1.949325489 | 0.000153634110798 | -0.047205241 | 0.969591912161 | 1.872711136 | 0.00233791578186 |
| peroxidase | TRINITY_DN34988_c0_g2 | E1.11.1.7 | 2.53805498 | 0.060622462 | 3.301319517 | 0.0149674 | 0.763182019 | 0.484628788 |
| peroxidase | TRINITY_DN39436_c0_g1 | E1.11.1.7 | 1.354009219 | 0.67406261 | 3.084139565 | 0.013683278 | 1.754675773 | 0.600858923 |
| hypothetical protein | TRINITY_DN4072_c0_g1 | E1.11.1.7 | -3.303331876 | 0.00273856028128 | -2.150368986 | 0.00388709231728 | 1.151478417 | 0.342218931986 |
| Plant peroxidase | TRINITY_DN4423_c0_g1 | E1.11.1.7 | -3.506563659 | 8.95672880888E-14 | -1.086903775 | 0.0597634440681 | 2.393434405 | 3.87755797901E-6 |
| peroxidase | TRINITY_DN4423_c1_g1 | E1.11.1.7 | -1.723853959 | 1 | 3.436277162 | 0.234968529 | 5.162277569 | 0.03672356 |
| hypothetical protein | TRINITY_DN5162_c0_g1 | E1.11.1.7 | 1.722966819 | 0.0325581226143 | 1.489302435 | 0.110106607878 | -0.247698101 | 0.796866279931 |
|  | TRINITY_DN53474_c0_g1 | E1.11.1.7 | 6.01445983 |  | 8.505967787 |  | 2.524307452 |  |
| hypothetical protein | TRINITY_DN5436_c0_g1 | E1.11.1.7 | -1.678681695 | 9.74661907771E-5 | -1.046828474 | 0.0919842492253 | 0.607049852 | 0.414783696846 |
| peroxidase | TRINITY_DN59628_c0_g1 | E1.11.1.7 | 4.160536632 | 0.135497303 | 6.579843656 | 0.00062543 | 2.445829636 | 1 |
| hypothetical protein | TRINITY_DN7038_c0_g2 | E1.11.1.7 | 1.503537121 | 0.011365254607 | 1.144291549 | 0.0518252078033 | -0.375392533 | 0.622123263519 |
| Plant peroxidase | TRINITY_DN746_c0_g1 | E1.11.1.7 | -3.525985502 | 1.13710663583E-39 | -2.102927358 | 1.52759204976E-11 | 1.400816728 | 1.79350553532E-5 |
| Plant peroxidase | TRINITY_DN746_c0_g2 | E1.11.1.7 | -4.149576092 | 3.59613355512E-14 | -2.696447285 | 6.85653585596E-18 | 1.424361542 | 0.000127186511164 |
| hypothetical protein | TRINITY_DN7525_c0_g1 | E1.11.1.7 | -1.602114397 | 2.11296356228E-6 | -0.715207126 | 0.121250223824 | 0.865901181 | 0.00393170611323 |
| hypothetical protein | TRINITY_DN8811_c1_g1 | E1.11.1.7 | -1.33323283 | 0.00143132872199 | -0.593650053 | 0.26039977591 | 0.723071887 | 0.18189781621 |
| cationic peroxidase 2-like | TRINITY_DN9950_c0_g1 | E1.11.1.7 | -4.440288939 | 0.0269092830158 | -1.002653892 | 0.615661807723 | 3.424838845 | 0.141594339388 |
| caffeoyl-CoA O-methyltransferase | TRINITY_DN2492_c0_g1 | E2.1.1.104 | -2.347882752 | 2.53788243049E-12 | -2.101252463 | 0.0837455735807 | 0.235368854 | 0.8956094648 |
| hypothetical protein | TRINITY_DN17922_c0_g1 | E2.3.1.133, HCT | -4.273821164 | 0.000843253484108 | -1.411825868 | 0.348079966789 | 2.842803058 | 0.157824311362 |
| hypothetical protein | TRINITY_DN2597_c0_g2 | E2.3.1.133, HCT | 2.434739259 | 1.2041297635E-20 | 1.881085853 | 6.66921508693E-13 | -0.568848443 | 0.0620203808765 |
| Transferase family | TRINITY_DN2597_c2_g2 | E2.3.1.133, HCT | -0.506988703 | 0.436993188 | -1.360247535 | 0.041099354 | -0.868249113 | 0.180446283 |
| hypothetical protein | TRINITY_DN13882_c0_g1 | E2.3.1.133, HCT;HHT1 | -3.757717663 | 1.66261145076E-15 | -3.141889213 | 1.7458408739E-5 | 0.581506843 | 0.680522006925 |
| Glycoside hydrolase | TRINITY_DN119_c0_g1 | E3.2.1.21 | -1.134679535 | 2.27248176796E-6 | -0.502729468 | 0.255316707492 | 0.608214455 | 0.120708090314 |
| Glycoside hydrolase | TRINITY_DN13980_c0_g1 | E3.2.1.21 | -4.010776202 | 4.96694975513E-10 | -2.587494413 | 4.07117689333E-10 | 1.396008964 | 0.00403517984172 |
| hypothetical protein | TRINITY_DN15122_c0_g2 | E3.2.1.21 | -3.43043497 | 0.030192242069 | -1.830432444 | 0.235543048612 | 1.581669973 | 0.547933774438 |
| hypothetical protein | TRINITY_DN1961_c2_g1 | E3.2.1.21 | -1.232825235 | 2.03223162998E-5 | 0.140408097 | 0.812698775464 | 1.362187281 | 0.000343163154834 |
| hypothetical protein | TRINITY_DN33937_c0_g1 | E3.2.1.21 | 2.247877673 | 1.45974907852E-12 | 1.507476895 | 0.00292841459586 | -0.754843336 | 0.0583743531476 |
| hypothetical protein | TRINITY_DN4279_c0_g1 | E3.2.1.21 | -1.779029303 | 0.00139179178397 | -2.322537777 | 0.000291939150254 | -0.567667147 | 0.634265197267 |
| hypothetical protein | TRINITY_DN427_c0_g1 | E3.2.1.21 | -1.704232192 | 5.41041550041E-6 | -0.829133343 | 0.116009390103 | 0.851225678 | 0.00306173429088 |
| Glycoside hydrolase | TRINITY_DN427_c0_g2 | E3.2.1.21 | 1.571355642 | 0.00165709473777 | 3.347135731 | 5.12587732794E-14 | 1.750833746 | 2.15449229553E-7 |
| Glycosyl hydrolase family 1 | TRINITY_DN6040_c0_g1 | E3.2.1.21 | -0.962882799 | 0.001008433 | 0.95512438 | 0.00431466 | 1.90611263 | 1.45E-09 |
| beta-glucosidase | TRINITY_DN8778_c1_g1 | E3.2.1.21 | -3.351174028 | 0.000890254954322 | -2.756329452 | 0.00373865338361 | 0.569878227 | 0.818734223552 |
| Glycosyl hydrolase family 1 | TRINITY_DN8_c0_g3 | E3.2.1.21 | -4.58612614 | 0.00404280267756 | -2.520609231 | 0.0164186094907 | 2.02979511 | 0.248829309321 |
| hypothetical protein | TRINITY_DN43691_c0_g1 | REF1 | -1.574544318 | 1.77857622487E-8 | -0.305307018 | 0.53717167049 | 1.252895402 | 0.0001666277059 |
| hypothetical protein | TRINITY_DN13204_c0_g1 | TOGT1 | 1.701208166 | 1.55312159542E-16 | 1.033495195 | 0.000708088240083 | -0.686539838 | 0.00786931930965 |
| hypothetical protein | TRINITY_DN185_c0_g1 | TOGT1 | 1.839924062 | 2.80421013843E-9 | 0.847586434 | 0.0647352950043 | -1.009404938 | 0.00250344073526 |
| hypothetical protein | TRINITY_DN9059_c0_g1 | TOGT1 | -1.882649332 | 1.70626896546E-13 | -0.732345436 | 0.037107040982 | 1.127927294 | 5.75333270714E-5 |
| hypothetical protein | TRINITY_DN9768_c0_g2 | UGT72E | 1.057256029 | 8.23233699061E-6 | 0.489248519 | 0.162018849382 | -0.58120186 | 0.0118490728566 |

Con is control, Salt (150 mmol/L Na+), and Salt+5-AzaC (200 μmol/L 5-AzaC + 150 mmol/L Na+), FDR<0.05000 indicates significant.
