# Supplementary material for: Enhancing effect of 5-azacytidine on saline–alkaline resistance of Akebia trifoliata and underlying physiological and transcriptomic mechanisms
Source: PeerJ. 2025 May 14;13:e19285. doi: 10.7717/peerj.19285 (PMC12085116; doi:10.7717/peerj.19285)
Supplement: Supplemental Information 3 [file peerj-13-19285-s003.doc]

**Table S3 Differential expression results of Photosynthesis**

| gene name | Gene ID | KO name | Salt vs Con | | Salt + 5-AzaC vs Con | | Salt + 5-AzaC vs Salt | |
| --- | --- | --- | --- | --- | --- | --- | --- | --- |
|  |  |  | Log_2_FC | FDR | Log_2_FC | FDR | Log_2_FC | FDR |
| hypothetical protein | TRINITY_DN1678_c2_g3 | ATPF0B, atpF | -1.490896771 | 8.67840904951E-12 | -0.304725922 | 0.330941039001 | 1.170517451 | 4.76177009084E-6 |
| hypothetical protein | TRINITY_DN501_c1_g9 | ATPF0B, atpF | 1.876840799 | 2.59771018418E-5 | 1.398486099 | 0.00606754817786 | -0.490029124 | 0.339772308099 |
| ATP synthase alpha/beta family | TRINITY_DN3498_c0_g1 | ATPF1A, atpA | 0.365883751 | 0.143130967 | 1.030135749 | 7.87408999687E-5 | 0.651931488 | 0.004703542 |
| ATP synthase delta chain | TRINITY_DN7461_c0_g1 | ATPF1D, atpH | -1.903671768 | 1.45597371866E-17 | -0.528396244 | 0.242149783383 | 1.363608775 | 8.75216913468E-5 |
| ATPase | TRINITY_DN19218_c0_g1 | ATPF1G, atpG | -1.321418879 | 0.0161366537355 | -0.654664307 | 0.428715647483 | 0.650115105 | 0.392528573597 |
| ATPase | TRINITY_DN6529_c0_g1 | ATPF1G, atpG | -2.846079016 | 2.82249955527E-5 | -1.489200824 | 0.588578078595 | 1.339959886 | 0.463479732323 |
| ATPase | TRINITY_DN7055_c0_g1 | ATPF1G, atpG | -1.708331498 | 9.79625290406E-13 | -0.30037567 | 0.625091794221 | 1.399490545 | 0.000623708024485 |
| cytochrome f | TRINITY_DN8624_c0_g4 | petA | 3.048558746 | 0.000345374478751 | 2.469371501 | 0.0310798204954 | -0.586560792 | 0.397097272174 |
| plastocyanin | TRINITY_DN5440_c1_g1 | petE | -1.697912338 | 9.02765290442E-12 | -0.209379998 | 0.741685362757 | 1.476143797 | 0.000103255235942 |
| ferredoxin-like | TRINITY_DN10861_c0_g3 | petF | -1.844045719 | 1.81621921909E-16 | -0.670521524 | 0.0578602326002 | 1.158479678 | 9.76501883946E-5 |
| ferredoxin | TRINITY_DN10861_c1_g1 | petF | -2.374020707 | 5.01370274897E-22 | -0.913803682 | 0.0339835765922 | 1.444728516 | 2.34508280332E-5 |
| Ferredoxin | TRINITY_DN23066_c0_g2 | petF | -1.925287996 | 4.91879506867E-10 | -0.901475651 | 0.0189130205009 | 1.003258922 | 0.000267745162092 |
| ferredoxin | TRINITY_DN48235_c0_g1 | petF | -1.572232917 | 1.73385429228E-10 | -0.248223378 | 0.684460708739 | 1.311644595 | 0.00060168240778 |
| Ferredoxin c 1 protein | TRINITY_DN7447_c0_g1 | petF | -2.05781683 | 3.65556726763E-16 | -0.605227975 | 0.226149870012 | 1.437108329 | 1.84999015849E-5 |
| cytochrome b6-F complex subunit 5 | TRINITY_DN3697_c0_g2 | petG | 1.273938153 | 0.0393630066619 | 0.975441276 | 0.164952332569 | -0.306282782 | 0.678974307557 |
| hypothetical protein | TRINITY_DN10032_c0_g1 | petH | -1.275571508 | 5.1820323725E-9 | -0.519358044 | 0.114248830578 | 0.743527689 | 0.0137353835004 |
| hypothetical protein | TRINITY_DN3614_c0_g1 | petH | -1.063212257 | 8.26680319328E-7 | -0.331908006 | 0.376900550162 | 0.719569189 | 0.0174604880936 |
| Photosystem I psaA/psaB protein | TRINITY_DN813_c0_g1 | psaA | 0.747655624 | 0.015626573 | 1.468555881 | 5.74389152511E-7 | 0.708310395 | 0.005854612 |
| hypothetical protein | TRINITY_DN1995_c0_g1 | psaD | -2.59058649 | 2.75216815204E-23 | -1.241338211 | 0.000985385090236 | 1.335262595 | 0.000402233698654 |
| hypothetical protein | TRINITY_DN593_c0_g1 | psaE | -1.159320293 | 1.95459376086E-6 | -0.182997518 | 0.661542278125 | 0.957366757 | 0.0001666277059 |
| Photosystem I PsaE | TRINITY_DN957_c0_g1 | psaE | -1.411903366 | 1.37846402043E-8 | -0.394220859 | 0.295248917993 | 1.000952878 | 0.00134062419199 |
| hypothetical protein | TRINITY_DN858_c0_g2 | psaF | -1.28612496 | 2.68183645767E-8 | -0.290157957 | 0.437033604483 | 0.982095996 | 0.00120459848112 |
| Photosystem I PsaG/PsaK protein | TRINITY_DN3011_c1_g1 | psaG | -2.00246915 | 1.84223510049E-15 | -0.702082212 | 0.0888940805786 | 1.286106078 | 0.000328574677119 |
| photosystem I reaction center subunit V | TRINITY_DN8870_c0_g1 | psaG | -3.336900597 | 4.87474484817E-7 | -1.40031402 | 0.00190119313296 | 1.913507104 | 2.64575957319E-7 |
| hypothetical protein | TRINITY_DN12980_c0_g1 | psaH | -1.43472081 | 3.14195261197E-10 | -0.176490815 | 0.691409810578 | 1.241889328 | 5.37170389984E-6 |
| Photosystem I reaction center subunit psaK | TRINITY_DN4818_c0_g1 | psaK | -2.00167801 | 6.60137432523E-16 | -0.600654291 | 0.113590307205 | 1.382669966 | 4.9055184042E-7 |
| Photosystem I PsaL | TRINITY_DN4136_c1_g1 | psaL | -2.481514002 | 3.88596852073E-22 | -0.804914686 | 0.0555691598534 | 1.659199712 | 2.74110514313E-7 |
| Photosystem I subunit O like | TRINITY_DN19066_c0_g1 | psaO | -1.674187334 | 4.18714167386E-10 | -0.728158622 | 0.0426398176726 | 0.928463916 | 0.00771247644786 |
| hypothetical protein | TRINITY_DN6003_c0_g1 | psb27 | -1.698120019 | 3.47561165064E-12 | -0.115767958 | 0.860916158194 | 1.567846034 | 3.64954955963E-6 |
| hypothetical protein | TRINITY_DN19712_c0_g1 | psb28 | -2.062547787 | 2.81333137313E-17 | -0.438984194 | 0.373739046009 | 1.609918173 | 3.79319670666E-6 |
| hypothetical protein | TRINITY_DN1264_c3_g2 | psbA | -1.411674565 | 0.0113395949554 | -0.137595507 | 0.86416977594 | 1.254762094 | 0.0083620774896 |
| photosystem II protein D2 | TRINITY_DN4760_c0_g2 | psbD | 1.890464641 | 4.92574076058E-12 | 2.547402935 | 1.16977310609E-12 | 0.646920803 | 0.0108376871513 |
| Lumenal portion of Cytochrome b559 | TRINITY_DN991_c0_g3 | psbE | 0.652282489 | 0.309888781 | 1.211773017 | 0.0228418069329 | 0.541472487 | 0.26045917 |
| oxygen-evolving enhancer protein 1 | TRINITY_DN13501_c0_g1 | psbO | -1.56216667 | 1.24169489336E-8 | -0.811515335 | 0.0282684464413 | 0.737787659 | 0.0843419486757 |
| hypothetical protein | TRINITY_DN134_c1_g3 | psbP | -1.201997999 | 9.57681619225E-7 | -0.33797194 | 0.408049314038 | 0.850479167 | 0.0148395225171 |
| Photosystem II PsbQ | TRINITY_DN1941_c0_g1 | psbQ | -1.484746378 | 1.40915688543E-9 | 0.199896243 | 0.694578270677 | 1.670276522 | 2.5559690253E-7 |
| hypothetical protein | TRINITY_DN22193_c0_g1 | psbQ | -1.158125316 | 1.32539777548E-6 | -0.019034223 | 0.971336851761 | 1.124235296 | 9.88980520238E-5 |
| hypothetical protein | TRINITY_DN9131_c0_g1 | psbQ | -2.337927937 | 1.21364711419E-20 | -0.794515472 | 0.00686449235776 | 1.525315582 | 2.01247940319E-7 |
| Photosystem II 10 kDa polypeptide PsbR | TRINITY_DN594_c1_g2 | psbR | -0.902142789 | 7.30E-05 | 0.229446891 | 0.57781305392 | 1.117292743 | 8.02E-05 |
| photosystem II 10 kDa polypeptide | TRINITY_DN8697_c0_g1 | psbR | -1.436125926 | 6.60903431931E-10 | -0.58289055 | 0.133910503615 | 0.840871174 | 0.0177831037412 |
| Photosystem II family protein | TRINITY_DN13401_c0_g1 | psbS | -1.68584128 | 4.37518700883E-17 | -0.514717271 | 0.199386629112 | 1.159486072 | 0.00017002696098 |
| Photosystem II reaction center W protein | TRINITY_DN13395_c0_g1 | psbW | -1.841945161 | 2.17081352525E-16 | -0.430529471 | 0.27403663881 | 1.395366616 | 8.37538486737E-7 |
| hypothetical protein | TRINITY_DN368_c0_g1 | psbW | -1.697688427 | 8.49212430026E-10 | -0.457941282 | 0.357118784155 | 1.219978425 | 0.000192712685901 |
| Photosystem II PsbY | TRINITY_DN8975_c0_g1 | psbY | -2.577369337 | 5.01798552503E-23 | -1.421375007 | 9.33015560306E-5 | 1.142620705 | 0.00354155829844 |
| Photosystem II PsbY | TRINITY_DN8975_c0_g2 | psbY | -2.571673638 | 3.69824232057E-24 | -1.076579694 | 0.00433560437243 | 1.479328055 | 1.81394755577E-5 |

Con is control, Salt (150 mmol/L Na+), and Salt+5-AzaC (200 μmol/L 5-AzaC + 150 mmol/L Na+), FDR<0.05000 indicates significant.
